# Perceptions of autism and experiences of stigma among Somali and Eritrean immigrant parents in Norway: a qualitative study

**DOI:** 10.3389/frcha.2026.1688576

**Published:** 2026-04-10

**Authors:** Abdi Gele, Hodan A. Duale

**Affiliations:** 1 Department of Health Service Research, Norwegian Institute of Public Health, Skøyen, Oslo, Norway; 2 Department of Maternal and Child Health, Somali Institute for Health Research (SIHR), Hargeisa, Somaliland

**Keywords:** autism, Eritrean, immigrants, knowledge, Norway, parents of children with autism, Somali

## Abstract

**Background:**

Autism is a major public health problem among immigrants, with immigrants from Somalia and Eritrea having one of the highest prevalence of autism in Norway. This study explored knowledge of autism and the experiences of stigma and discrimination among Somali and Eritrean parents of children with autism.

**Methods:**

An in-depth interview was used to collect data from 15 Somali and Eritrean parents of children with autism. We used a pilot-tested semi-structured interview-guide. A mixed of convenience and snowball sampling was used to recruit participants who 1) identified as an Eritrean or Somali immigrant, 2) identified as a parent of a child with autism, and 3) were 18 years and older. The interviews were recorded, transcribed verbatim, translated, and cleaned for errors. A thematic analysis was used to analyze the data using Nvivo-14 software.

**Results:**

The findings showed that Somali and Eritrean parents had relatively good knowledge of the risk factors for and treatment of autism. Some parents had feeling that the MMR vaccine was behind the autism of their children. Similarly, five of the parents reported that bone marrow from camels can help alleviate the symptoms of autism. Widespread stigma, judgment and blaming of parents and children with autism were reported. This was attributed to a lack of knowledge about autism among Somali and Eritrean communities in Norway.

**Conclusion:**

Community-based initiatives that engage key stakeholders including civil society organizations, can help to address misconceptions about autism. Furthermore, national and community-owned mass media should produce programs in which individuals with ASD and their families share their experiences and struggles and health professionals provide accurate information about the disorder while promoting inclusion and acceptance.

## Background

Autism spectrum disorder (ASD) is a developmental disability caused by differences in the brain, leading to problems in social communication, interaction and restricted or repetitive behaviors or interests (1). There is an evidence that ASD diagnosis may be more common among children with at least one immigrant parent than among children with two nonimmigrant parents in Europe (2–4). The number of migrants and their descendants are expanding as the number of migrants has increased substantially in the last decade (5), reaching 1 in 5 people in Norway in 2023. A migrant is an individual who has resided in a foreign country for more than one year (5). In addition, second-generation migrants who are persons born in the country with at least one parent born abroad (6).

Research has shown that culture shapes the way families, communities and professionals comprehend and treat developmental disorders (7, 8). The interpretations of the causes and treatments of autism are particularly susceptible to the influence of culture because there are no universally agreed-upon causes for autism, while its diagnosis relies largely on behavioral criteria, which vary considerably across cultures (7, 9). While Western biomedicine considers ASD to be affected by genetics and environmental factors (10), the absence of agreed-upon, scientifically valid treatments increases the likelihood that treatment decisions are based on cultural and local beliefs and values (7, 11). Immigrant families are largely connected with their native culture and networks, and this connection may shape their perception of autism. For example, eye contact of children with older people or authorities is traditionally seen as disrespectful in some cultures (12). Although this may not be the culture of Somali and Eritrean people, it is important to understand how atypical autism symptoms may be perceived differently in different cultures including the mainstream culture. Research on immigrant parents has shown that parents' perceptions of autism in their children are influenced both by their native culture and by the biomedical and other health-related narratives in their host country (13). Because of its unique sociocultural environment, immigrants from Africa may experience divergent conceptions of ASD than immigrants from other cultures (14).

Nonetheless, ethnic minorities in Europe are severely underrepresented in autism research (15). Therefore our knowledge of symptoms as well as the screening and diagnostic instruments for the identification of ASD and the interventions that support people with ASD are likely to be culturally biased (16). Nevertheless, a prior systematic review of seventeen studies concerning autism among immigrants in Europe revealed that fifteen out of the seventeen studies highlighted a greater prevalence of ASD among immigrant children than among native children (17). Moreover, a study in the UK reported that children with mothers of black ethnicity have an over twofold greater risk of autism than do mothers of white ethnicity (18). The study concluded that “maternal immigration is associated with substantial increased risk of ASD with differential risk according to different regions of birth and possibly ethnicity” (18).

Many studies exist among migrants from Somalia and Eritrea in Europe and the USA (2–4, 19–23). Hewitt et al. reported that the prevalence of ASD was greater in White and Somali children in Minneapolis than in Black and Hispanic children (24). Similarly, a study in Sweden showed that the prevalence of autism was between four and five times greater in children of Somali background than in those of non-Somali origin (25). Although Somali refugees and immigrants have high rates of ASD, Somalis call autism the “Western disease” given the absence of the word “autism” in the Somali language, and absence of healthcare for autism in Somalia (26). These findings are supported by a recent study in Sweden showing that Somalis experience higher rates of ASD and that they consider autism a “Western disease” that only affects Somali children in the diaspora (23). As parents are the first to recognize signs and symptoms of autism, research shows that parents' knowledge and experience will affect the diagnosis, treatment, and prognosis of children with ASD (23). Previous research revealed an association between discrimination and refraining from seeking medical treatment (27). This study explored knowledge of autism and the experiences of stigma and discrimination among Somali and Eritrean parents of children with autism.

## Methods

### Study participants

We used a qualitative design, which is appropriate for describing, explaining, and analyzing complex phenomena that have not been explored well. Fifteen in-depth interviews (IDIs) with immigrant parents of children with autism were conducted in five different cities across Norway. The interviews were held between November 2023 and February 2024. The participants were born in either Somalia or Eritrea, except one mother who born in Norway by two East Africa born parents. We included parents of children who were diagnosed with autism by a doctor, while we excluded parents of children who had other developmental disorders. The aim was to explore Somali and Eritrean parents' knowledge of autism, and their experiences with stigma and discrimination.

### Recruitment and data collection

A total of 15 parents were recruited for the study following information about the study that was shared through Facebook and community meeting centers. Nine of the participants contacted the researcher directly to confirm their willingness to participate in the study, while the remaining six participants came through snowballing. We used a question guide with semi-structured questions for data collection. The interview questions were generated to formulate a series of open-ended questions that elicited a response from the participants about their experiences and understanding of the causes and treatment of autism. We adopted a coproduction approach in which two parents with autistic children were involved in the initiation of the discussion about the recruitment of participants, the data collection, and the write-up of the results. Coproduction research involves collaboration between researchers and end-users of the research to identify research questions, design and priority setting, codelivery of research activities, and involvement in knowledge exchange (28). Before starting the data collection, information about the study was provided to the participants in different venues based on each participant's preference. Through an information letter, the researcher explained the study objectives, benefits and risks associated with the study to each participant to promote transparency. The interviews were conducted in Somali for Somalis by the first author, who is originally from Somalia and is a qualitative researcher, and in Tigrinya for Eritreans by a pre-trained research assistant with masters in health science who was born in Eritrea. Twelve interviews were conducted via telephone, while three interviews were conducted face-to-face at locations selected by the participants. The interview process was concluded when it became evident that the data no longer produced novel codes or themes. Iterative coding and constant data comparison demonstrated that ongoing data collection yielded redundant information, confirming that saturation had been reached. This understanding supports the adequacy of having a sample of 15 participants, as it aligns with the principle of data saturation in qualitative research, where additional data no longer reveals new insights (29). We ensured robustness and rigor throughout the research process, thereby facilitating a comprehensive understanding and the extraction of meaningful insights. The interviews were audio recorded and transcribed verbatim by the interviewers.

During the study process, we considered ourselves as insiders as we were immigrants from the same communities, while at times we considered ourselves as outsiders given our position as researchers, in addition to not having practical experience of caring a child with autism. However, we acknowledge that being both insiders and outsiders during the study process presented its own challenges and limitations. As insiders, we had a deeper understanding and connection to the community and were able to build trust and rapport with the participants. However, this also meant that there was a potential for bias in our research due to our personal experiences and perspectives. As outsiders, we were able to maintain a level of objectivity and distance, but this also meant that we may have missed out on important insights and nuances that only insiders may have observed. Ultimately, this dynamic of being both insider and outsider during the study process allowed us to take a more comprehensive and nuanced approach to our research. It also served as a constant reminder to critically analyze our own biases and perspectives in order to accurately capture and understand the experiences of the participants. Overall, this unique perspective helped to strengthen the validity and reliability of our research findings.

### Data analysis

The data were analyzed using thematic analysis; a method that describes the material collected and explores its meaning to reflect what the participants said in the most objective and reliable way (30). Twelve interviews were transcribed in Somali, while three interviews that were taken in Tigrinya, were transcribed in Norwegian. The reason was that the Eritrean research assistant could speak fluent Norwegian, and very good English, but it was quicker for her to write the transcripts in Norwegian than English. Finally, all the transcripts were translated to English using forward translation by the authors who speak Somali, English and Norwegian. We preferred forward translation given the competence of interviewers and authors in the local culture and the language used for the interviews (31).

To analyze the data, we employed thematic analysis as outlined by Braun and Clarke (32). We began by closely reviewing the interview transcripts to deeply understand the data and immerse ourselves in the parents' narratives. We then generated initial codes to identify key patterns and recurring topics related to their experiences. These codes allowed us to organize the data into meaningful segments. Next, we meticulously examined these codes to identify overarching themes that captured the essence of the parents' lived experiences regarding stigma. Once the themes were clearly defined, we conducted a comprehensive review to ensure they truly reflected the data and made adjustments as needed. Finally, we refined and named the themes, providing detailed descriptions that effectively convey the insights gathered from the parents' stories. Throughout this process, we maintained a systematic approach, ensuring a thorough and insightful analysis. We used NVivo software (version 14) for the analysis of the data.

A wide range of experiences and perspectives from the diverse in-depth interviews were verified against each other, to help ensure the trustworthiness of the study results. Following the recommendation of employing a minimum of two methods of rigour within qualitative research (33), we conducted member checking, which is a practice to increase study credibility and confirmability that involves asking a research subject to verify the transcription of an interview (34). We asked the participants to verify the completeness and accuracy of the transcript to ensure that the transcript truthfully reflected the meaning and intent of the participants' viewpoints. We also conducted investigator triangulation and reflexivity to ensure the scientific rigour of the current study (35). Two investigators were involved in the process of data analysis, which allowed different perspectives and ideas. We received ethical approval from the Norwegian Institute of Public Health (eProtokollnr: 4192-4192). Full information about the study was given to participants prior to data collection. Participants were informed that they could withdraw from the study at any time. Participants' attitudes toward ethical conduct are vital to a relationship likely to produce high-quality data (29). During the research process, we ensured the confidentiality of participants by presenting the results in a manner that prevents any identification of individual participants.

## Results

Of the 15 participants, 12 were mothers (Table 1). The 16 children with autism they cared for were 4–24 years old, and two were girls. All the children with autism were born in Norway. Seven of the parents had a university education, four had a secondary education, three had a primary education, and one had no formal education.

**Table 1:** Characteristics of study participants and their children.

Parents' characteristics				Their children's characteristics				
N	Relation	Education	Born	N-Children	Born	Diagnosed with ASD	Age of the child with ASD	Sex
1	Mother	Secondary	Somalia	6	Norway	2	8 &14	Boys
2	Mother	University	Norway	2	Norway	1	5	Boy
3	Mother	University	Somalia	2	Norway	1	8	Boy
4	Mother	University	Somalia	3	Norway	1	4	Boy
5	Mother	University	Somalia	3	Norway	1	20	Boy
6	Mother	University	Somalia	5	Norway	1	14	Boy
7	Mother	Secondary	Somalia	3	Norway	1	6	Boy
8	Mother	Secondary	Somalia	4	Norway	1	6	Boy
9	Mother	No education	Somalia	2	Norway	1	17	Boy
10	Mother	Secondary	Somalia	4	Norway	1	17	Boy
11	Father	University	Eritrea	3	Norway	1	9	Girl
12	Father	Primary	Eritrea	8	Only 1 born in Norway	1	6	Girl
13	Father	Primary	Eritrea	2	1 born in Norway	1	6	Boy
14	Mother	University	Somalia	2	Norway	1	7	Boy
15	Mother	Primary	Somalia	2	Norway	1	24	Boy

As shown in Table 2, three themes and 11 subthemes emerged from the analysis. The themes included suspected causes of autism, presumed treatment, discrimination and stigma. Unlike the participants of our recent study in Somalia (36), most Somali and Eritrean immigrants in this study presented biomedical factors as the causes of autism. The reported causes of autism were categorized as vaccine, stress during pregnancy, preterm birth and complications during pregnancy, lack of essential vitamins, and older age of the parents (Table 2).

**Table 2:** Themes and categories that emerged from the interviews.

Themes	Categories	N of participants contributing (*N* = 15)	N of transcript excerpts assigned
Suspected causes	Vaccine	4	6
	Stress	6	21
	Preterm and complication during birth	3	4
	Lack of essential vitamins	3	9
	Older age	1	2
Treatment	Bone marrow from camels	5	7
	No treatment for autism	7	13
Discrimination	Stigma	15	23
	Blaming	12	21
	Judgment	4	9
	Awareness to reduce stigma and discrimination.	14	18

### Vaccine

Some participants believed that vaccines, particularly the Measles, Mumps, and Rubella (MMR) vaccine, are potential causes of autism in their children. One participant was uncertain which vaccine to blame but ambivalently mentioned the vaccine that children receive at 13 months. The belief that vaccines cause autism has hindered some participants from vaccinating their children with the MMR vaccine.

When my child was from 6 months to 1 year, he used to focus things, had eye contact, and could say mamma, pappa, etc., but when he received the vaccine, he stopped everything, and before the vaccine he was fine, all his problems began at the time of the vaccine. Everything happens with Allah's will, but I suspected the vaccine. I had no family history of autism, so I cannot think of other issues. (Participant 7)

My child could say words when he was one year old. He used to talk less than other children, but he was pronouncing the few words he knew very clearly. He ceased to talk after he received the vaccine, that is, when his problem began. (Participant 9)

A vaccine hesitant mother stated that there is a link between the MMR vaccine and autism, and she admitted that her children had not taken any vaccine, yet she had a child with autism.

I am a very cautious person. I didn't give that vaccine to my children. There are many women who associate the autism of their children with the MMR vaccine. My child didn't take that vaccine. My family does not have autism either. I am against vaccines, my children didn't take swine flu vaccine either, I don't give vaccines to my children … I use natural stuff such as vitamins instead of vaccines. (Participant 5)

### Stress

Most participants attributed the cause of the autism of their children to stress during pregnancy. They mentioned that they were experiencing stressful situations during the pregnancy of the child with autism. Most of them mentioned domestic problems involving family break-up, separation, or other serious domestic issues during pregnancy. They clearly stated that they did not experience such a level of stress when they conceived other nonautistic siblings.

I was in a very stressful situation when I became pregnant with my child. Stress during pregnancy is the main factor for having autistic children. I asked many mothers about the difference between the child with autism and other siblings during pregnancy, and every mother told me that she experienced stress during the pregnancy of the autistic child. Every woman I talked to agreed that the reason was stress. (Participant 1)

I had a family problem. I was studying at the university at that time. I became isolated, and I got pregnant. The pregnancy of the autistic child was different from that of the other two children with regard to the level of stress I experienced. I had to submit my bachelor's thesis within a short deadline, plus pregnancy plus family problems (Participant 4)

#### Preterm and complication during birth

Preterm birth coupled with prolonged labor, falling on the ground in the second trimester and the use of forceps were reported by some participants. One participant shared her thoughts about the forceps that were used to assist the delivery, and subsequent asphyxia, which she thought might be the reason behind her child's autism.

I think my child was born with autism, he was born preterm, and I had difficult delivery. He was deprived of oxygen during labor; if that didn't harm my child, I couldn't think anything else. He was born a month earlier than the term, the water broke, and I was taken to the hospital. I was given medicine to accelerate the labor, the child did not come down toward the cervix, he was helped out using forceps, and he got some injuries on the head. He even lost some hair because of that … if that didn't hurt him, I cannot think anything else …  (Participant 3)

I was new to Norway, I didn't know the environment, I was not careful of walking on slippery ground. Then, I fell down when I was 5 months pregnant. I started bleeding, and the child was born at the 6th month, so I think it is the damage caused by the fall. (Participant 15)

#### Lack of essential vitamins

Parents stated that ASD could be passed from parents to the unborn child. However, parents associated the autism of their children with deficiency of essential vitamins such as vitamin D and other vitamins.

I think there are many factors that can cause autism in my child: I lacked vitamin D, may be the food I was eating when pregnant where not nutritious, and it can be genetic too. Although my family does not have autism nor my husband's family as far as we know, but they can have undiagnosed autism. (Participant 2)

When I was pregnant, I traveled abroad, so I became sick during travel; I lacked vitamins, so I think that may be the cause. (Participant 6)

I suspected two issues … stress and deficiencies of important vitamins such as vitamin D and iron … (Participant 8)

#### Older age

One participant reported the “older age” of parents as a potential cause of autism, without explaining the underlying mechanisms. The participants mentioned that they were both over 40 years of age when the mother conceived the child with autism.

The reason can be, as I said earlier, to have a baby at an older age. I and my wife are both old adults, and we have the child with autism over the age of 40. Therefore, I think that old age could be the major reason for having a child with autism. (Participant 12)

### Theme 2: treatment

While almost all participants believed that the different therapies that their children receive from the health system are the best available treatment for their children's autism, they also mentioned traditional treatment, which they believe can help improve the symptoms of autism. The traditional treatment highlighted by most participants was bone marrow from camels. Several participants also stated that autism cannot be cured, while only one participant insisted that autism can be cured like any other disease.

#### Bone marrow from camels

Some parents of children with autism believe that bone marrow is nutritious; thus, it can improve the health of children with autism. Parents believe that bone marrow can be used as an adjunctive treatment but is not the main treatment, as they believe in the health system. One of the participants, who also distributes bone marrow, reported that her children's situation improved significantly when she started using bone marrow and that many mothers of children with autism who received bone marrow from her store provided very positive feedback. According to parents, the born marrow is prepared in the form of butter and then mixed with food for the child.

I gave bone marrow to my child. I mix it with food. He began to sleep well. Earlier, he used to chew the duvet and had problems sleeping. After giving him bone marrow from a camel, he improved a lot in different ways. He became calm, slept well, etc. I shared the information to my network, and all mothers told me that their child with autism had made a significant improvement after he consumed the bone marrow. The bone marrow is rich in collagen, which aids in brain development (Participant 1)

I took him to different places for help, I give him camel milk, people told me that bone marrow can help his condition. In Somalia, people used to give bone marrow to people who break their legs or arms for healing. It may also help the brain. His father told me to try bone marrow, I have received it now, so I will give him. (Participant 5)

#### No treatment for autism

Although some parents believed that bone marrow may help improve the autism of their children, the majority of the parents believed that autism cannot be cured but that adjunctive treatments may only improve the child's condition.

I don't think my child will recover from autism … when you have fever you take anti-pain, but I don't think autism can be cured that way. (Participant 3)

When I have realized that my child has autism, it was clear to me that the child will never be cured from this condition. My child does not have severe autism; he can communicate, but he has behavioral challenges. I don't believe he will be cured. (Participant 15)

### Theme 3. Discrimination against parents and children

All participants, regardless of their country of birth, mentioned that they experienced stigma, discrimination, and judgment from their communities. They mentioned varying degrees of stigma and discrimination, which, at times, prompted parents to avoid meeting places and participating in community activities. Some participants stated that they keep their children with autism away from playing with other children. To avoid discrimination, many parents emotionally shut themselves off and were unwilling to talk about their child with other community members. Parents attributed the discrimination they experienced to their community's lack of knowledge about autism.

### Stigma

Parents described how they were seen as “different” because of their children's autism. Almost all parents reported that people call them “women with a sick child” or fathers of the sick child. The stigma experienced by parents prompted many parents to lose interest in mingling with their community members or participating in community activities and socialization.

Our people treat parents with autistic children badly. The way people talk to us is truly depressing. They call us “mother with sick child”, her child is crazy, etc. There are many mothers who keep the autistic child at home to avoid being insulted and harassed by the people. Our people do not understand autism at all. (Participant 15)

Somali people stigmatize autism, and families keep children with autism at home. They do not take them outside to participate in social activities. They call us “families with sick children”. (Participant 1)

Parents of children with autism have limited contact with the community; they stay in isolation, do not go outside, think that people will embarrass them with the autistic child (Participant 11).

### Blaming

According to the participants, many people in our community believe that every child must act normally in public, and if not, this means that the child must be spoiled by the parents. Parents reported being blamed for their child's behavior, labelled to “bad parents” because people become upset with their child's restless behavior. Some parents experienced a situation in which other mothers tried to discipline the autistic child by thinking that the mother of the child did not give him proper upbringing regarding how to behave well.

People told me that I should discipline the child; they blame me of not showing him the right way that is why he is behaving nasty. They think he is a spoiled child, which I have to be blamed. (Participant 2)

Our people do not understand autism well. When I tell them that my child has autism, they give me 100 suggestions that, according to them, can make my child like any other normally growing child. When they see my child is acting differently, they tell him “Hey, listen to your mum, what is wrong with you?” … they think this is a spoiled child and he can be corrected. (Participant 8)

### Judgment

Participants reported that some community members believe that having a child with autism can only occur if the mother is cursed by her parents or by other people. This belief may sometimes be held by close family members such as brothers, sisters, parents and other close family members.

There are people who think that the reason why my child has autism is because I was cursed by my parents. (Participant 14)

A friend of mine said to me that “my aunt also got a child with autism because she was cursed by her parents”, implying that I am also cursed because I have a child with autism … even my father asked me about the mistake I have done to deserve a child with autism. They judge me, they ask me what has gone wrong …  (Participant 4)

People call me, “mother with sick child”-they say, what is wrong with her to have a child with autism, is she cursed? They try to find a reason. You lose interest in going to where Somalis meet. Our culture is rude to families of children with disabilities. (Participant 10)

### Awareness to reduce stigma and discrimination

The study participants attributed stigma and discrimination to the community's lack of knowledge on autism, and they underscored the importance of increasing public knowledge on autism. Awareness campaigns about autism among immigrants to reduce stigma and discrimination against parents of children with autism were suggested by study participants.

Somali people are among the largest group of people with autistic children, but they have the least knowledge about this condition. They need to have awareness campaigns to understand that there are children who are different from other children … (Participant 3).

## Discussion

While most of the parents in the study did not know the causes of autism, some parents stated biomedical causes such MMR vaccine. This result is in line with our recent findings in Somalia, where half of the parents associated the onset of autism with the measles vaccine (36). This finding also concords with other studies among Somalis in Europe and the USA (22, 23). The parents' concern about that link between childhood vaccination and autism is common, not only among immigrants, but also general population in North America (37, 38). We do not know whether the suspicion of MMR vaccination by immigrant parents is based on the co-occurrence of the timing of the vaccine and the manifestation of autism symptoms or may be influenced by the conspiracy resulting from the study published by Andrew Wakefield and his colleagues (39) in the *Lancet*. The Andrew's study suggested that the MMR vaccine may predispose children to behavioral regression and pervasive developmental disorders. The study prompted a sudden drop of MMR vaccination as parents were concerned about the risk of autism after vaccination (40). A prior studies in Sweden and Norway stated that Somali parents had low vaccine acceptance and delayed MMR vaccination because of the fear that children would “stop talking” and develop autism (41, 42). The immigrant parent's perception that autism may be caused by the measles vaccine can be counterproductive to global efforts to eradicate measles (42). A study found that the perception that MMR causes autism to Somali children is a fear-rumor that spread across the community members (23). A fear-rumor often reflect the anxiety among community members toward a situation (43). The transmission of the fear-rumor across Somali communities living in different continents is possibly motivated by their efforts to fact-finding of the causes of increasing prevalence of autism among their children (43). Therefore, to counteract this rumor and subsequently eliminate MMR vaccine hesitance among immigrant parents, a targeted awareness campaign is necessary.

Parents also associated severe stress during pregnancy with autism in their children. An epidemiological study had shown that prenatal stress may increase the likelihood of ASD, although it is not a definitive cause (44). In line with this finding, a large national registry study in Denmark reported an association between maternal bereavement and ASD (45), while another study in Denmark revealed that maternal psychiatric conditions were one of the strongest prenatal risk factors for ASD (46). In Sweden, a register-based study also revealed a relationship between 3rd trimester maternal stress exposure and the risk of ASD (47). The association between prenatal-maternal stress and the risk of ASD has also been supported by a meta-analysis (48). It is essential to understand that multiple factors contribute to the development of ASD, and stress is just one piece of the puzzle. The findings that most of the study participants had severe stress during pregnancy supports the prior findings that stress during pregnancy may be a risk factor for autism.

Labor complications, genetics, preterm delivery, vitamin D deficiency and older age of the parents were also mentioned by participants as causes of their children's autism. This finding is in line with the findings of a prior review on environmental risk factors for autism, which reported all those factors are risk factors for autism (49). For example, studies have shown that every 10-year increase in maternal and paternal age increases the risk of ASD in offspring by 18% and 21%, respectively (50). In contrast to our findings, the parents who participated in our recent study in Somalia believed that their children acquired autism as opposed to being born with it (36). The difference could be because nearly half of our study participants in Norway had a university education, many of whom studied the health field, which may explain why they had better knowledge of biomedical risk factors for autism than their counterparts in Somalia. Beliefs about the causes and risk factors for autism often shape attitudes toward ASD and its treatment (51). Misperception of the causes can lead to delays in seeking medical attention and reliance on alternative treatments. Because the majority of our study participants' beliefs regarding the causes of autism were in line with the risk factors stated in the literature, they are likely to trust the biomedical treatment provided to their children by the health system.

While most participants reported that autism cannot be cured, few participants reported that bone marrow driven from camels helps alleviate symptoms of autism in their children. An earlier case study reported that camel milk contributed to the successful management of autistic children's symptoms (52). The mother reported that “beginning at age 9, my autistic child drank one half cup of raw camel milk a day and experienced overnight an improvement in his symptoms” (52). Camel products including camel milk and camel meat are long considered a functional food for cures and remedy for many ailments including Asthma, autism and Crohn's disease in many cultures around the world (53). While there is no scientific evidence supporting the efficacy of camel bone marrow in treating autism, it is perceived by participants as worth trying in the hope that it may benefit their children. Various traditional societies often hold beliefs in the healing power of specific remedies for certain illnesses. However, without empirical validation, these beliefs remain unsubstantiated. In contrast to the participants in our study, almost all participants in a previous study in Somalia sought traditional treatments for autism, with some even seeking care from traditional healers who often persuade parents that their child is bewitched or possessed by a Jinn, conditions they claim can be remedied through traditional methods (36). The perceived link between autism and the MMR vaccine, coupled with the belief in camel bone marrow as worth trying, underscores the need for culturally tailored communication strategies. Misinformation around autism and its causes can perpetuate misunderstanding and hinder effective intervention.

All participants in our study reported widespread discrimination, stigma, isolation, blaming, labeling, and judgment. This finding is in line with the findings of our recent study in Somalia, where all respondents reported experiencing discrimination (36). This finding also concords with a study among Somalis in the UK in which one of the participants stated that “one of the reasons that people hide their children's disability, and their children with disability, is that they are worried that people are going to say   …  She's the one who has got a child with autism” (54). In line with our findings, this study also reported that children with autism were labeled and stereotyped as sick and naughty and that parents were blamed for not controlling them, leading to social rejection and isolation (54). When children are labeled naughty and parents are blamed for not disciplining them, the consequence could be social isolation of both parents and their children. Discrimination and stigma imply the experience of unfair treatment, discrediting, devaluing, and shaming of a person because of characteristics or attributes that they possess, which often leads to negative social experiences such as isolation (55). Despite this, stigma related to autism and its impact on families is a pervasive issue affecting various cultures and settings worldwide (56, 57). There are primarily two forms of stigma: felt stigma, which is internalized due to a fear of being subjected to external discrimination; and enacted stigma, which involves overt acts of exclusion or prejudice (56, 57). In line with our study, individuals with autism spectrum disorder (ASD) and their families can experience both types of stigma (56). When family members experience stigma because of their association with someone who has ASD, it is known as affiliate stigma (58). In a study conducted among caregivers in South Korea and among immigrants in Canada, it was observed that having a child with ASD could lead to adverse effects such as social marginalization (56, 59).

Certain factors influence the degree of stigma associated with autism, such as the quality and quantity of contact that autistic people and their caregivers have with others, as well as cultural understanding, such as the specific beliefs of communities about autism (60). A study among Somali immigrants in the UK reported lack of a Somali word for autism, which indicates that autism is less known among Somalis (61). Stigma often arises from a lack of understanding (62). Therefore, a lack of understanding of the Somali community about autism could be the reason behind the stigma subjected to families of children with autism. Our study participants reported that they were blamed and judged of having a child with autism. A prior study reported that families of people with mental illness experience shame because they are blamed for being responsible for the illness (63). In concordance with our study, a study showed that parents were blamed for their offspring having a mental illness, leading to feelings of shame (64). In general, ASD imposes a considerable emotional and physical burden on families, and when that emotional toll is exacerbated by discrimination, stigma and judgment, this double burden may lead to social isolation and exclusion (65). Nurjannah et al. noted that mental health stigma has negative implications for the human rights and wellbeing of patients and their families (66) while preventing parents from seeking diagnosis and treatment, which may undermine the health of children with ASD (67).

This study has both strengths and limitations. The 15 in-depth interviews with parents from Eritrea and Somalia, including both mothers and fathers, added substantial depth and richness to the results. The ages of children with autism ranged from 4 to 24 years old, but we did not explore how parental experiences with stigma and discrimination might vary based on the age of their children. This omission may have concealed important insights into how discrimination challenges differ across developmental stages. Additionally, the majority of study participants possessed a university education, indicating that the findings may not be entirely applicable to parents with lower levels of education or those lacking proficiency in Norwegian. Educational attainment often confers numerous advantages, and as such, the experiences and challenges reported by well-educated participants may substantially differ from those encountered by parents with less formal education. As a result, the study's findings may not comprehensively capture the diversity of experiences within the broader Somali and Eritrean communities in Norway, potentially limiting the applicability of these insights across all demographic segments within these populations.

## Conclusion

To our knowledge, this is the first study exploring the perceptions of Somali and Eritrean immigrants raising autistic children in Norway. Our research findings can help inform policymakers, service providers, community organizations, and advocacy groups to better understand and address the needs of Somali and Eritrean parents raising autistic children. To counteract stigma and discrimination against these families, developing and disseminating culturally tailored information that reflects the unique sociocultural contexts of Somali and Eritrean communities is needed. Community-based initiatives that engage civil society organizations and health institutions can help to disseminate information and address misconceptions about autism. Furthermore, national and community-owned mass media should produce programs in which individuals with ASD and their families share their experiences and struggles while health professionals should provide accurate information about the disorder while promoting inclusion and acceptance.

## Data Availability

The original contributions presented in the study are included in the article/Supplementary Material, further inquiries can be directed to the corresponding author/s.
